# The association of plasma biomarkers with computed tomography-assessed emphysema phenotypes

**DOI:** 10.1186/s12931-014-0127-9

**Published:** 2014-10-12

**Authors:** Brendan J Carolan, Grant Hughes, Jarrett Morrow, Craig P Hersh, Wanda K O’Neal, Stephen Rennard, Sreekumar G Pillai, Paula Belloni, Debra A Cockayne, Alejandro P Comellas, Meilan Han, Rachel L Zemans, Katerina Kechris, Russell P Bowler

**Affiliations:** Department of Medicine, National Jewish Health, 1400 Jackson St, Denver, CO 80206 USA; Department of Medicine, University of Colorado School of Medicine, Aurora, CO USA; Department of Biostatistics and Informatics, Colorado School of Public Health, University of Colorado Denver, Aurora, CO USA; Channing Division of Network Medicine, Brigham and Women’s Hospital and Harvard Medical School, Boston, MA USA; Cystic Fibrosis/Pulmonary Research and Treatment Center, Department of Medicine, University of North Carolina at Chapel Hill, Chapel Hill, NC USA; Department of Internal Medicine, Pulmonary, Critical Care and Allergy Division, University of Nebraska Medical Center, Omaha, NE USA; Hoffman La Roche, Nutley, USA; Genentech, Member of the Roche Group, South San Francisco, CA USA; Department of Internal Medicine, University of Iowa, Iowa City, IA USA; Department of Internal Medicine, Division of Pulmonary and Critical Care, University of Michigan Health System, Ann Arbor, MI USA; Current address: Eli Lilly and Company, Lilly Corporate Center, Indianapolis, IN USA; Current address: Boston Scientific, San Jose, CA USA

**Keywords:** COPD, Biomarkers, RAGE, ICAM1, CCL20, Emphysema

## Abstract

**Rationale:**

Chronic obstructive pulmonary disease (COPD) is a phenotypically heterogeneous disease. In COPD, the presence of emphysema is associated with increased mortality and risk of lung cancer. High resolution computed tomography (HRCT) scans are useful in quantifying emphysema but are associated with radiation exposure and high incidence of false positive findings (*i.e.*, nodules). Using a comprehensive biomarker panel, we sought to determine if there was a peripheral blood biomarker signature of emphysema.

**Methods:**

114 plasma biomarkers were measured using a custom assay in 588 individuals enrolled in the COPDGene study. Quantitative emphysema measurements included percent low lung attenuation (%LAA) ≤ −950 HU, ≤ − 910 HU and mean lung attenuation at the 15^th^ percentile on lung attenuation curve (LP15A). Multiple regression analysis was performed to determine plasma biomarkers associated with emphysema independent of covariates age, gender, smoking status, body mass index and FEV_1_. The findings were subsequently validated using baseline blood samples from a separate cohort of 388 subjects enrolled in the Treatment of Emphysema with a Selective Retinoid Agonist (TESRA) study.

**Results:**

Regression analysis identified multiple biomarkers associated with CT-assessed emphysema in COPDGene, including advanced glycosylation end-products receptor (AGER or RAGE, p < 0.001), intercellular adhesion molecule 1 (ICAM, p < 0.001), and chemokine ligand 20 (CCL20, p < 0.001). Validation in the TESRA cohort revealed significant associations with RAGE, ICAM1, and CCL20 with radiologic emphysema (p < 0.001 after meta-analysis). Other biomarkers that were associated with emphysema include CDH1, CDH 13 and SERPINA7, but were not available for validation in the TESRA study. Receiver operating characteristics analysis demonstrated a benefit of adding a biomarker panel to clinical covariates for detecting emphysema, especially in those without severe airflow limitation (AUC 0.85).

**Conclusions:**

Our findings, suggest that a panel of blood biomarkers including sRAGE, ICAM1 and CCL20 may serve as a useful surrogate measure of emphysema, and when combined with clinical covariates, may be useful clinically in predicting the presence of emphysema compared to just using covariates alone, especially in those with less severe COPD. Ultimately biomarkers may shed light on disease pathogenesis, providing targets for new treatments.

**Electronic supplementary material:**

The online version of this article (doi:10.1186/s12931-014-0127-9) contains supplementary material, which is available to authorized users.

## Introduction

Chronic obstructive pulmonary disease (COPD) is a phenotypically heterogeneous condition characterized by airflow limitation that is not fully reversible [1]. Some, but not all, COPD subjects have emphysema, *i.e.,* airspace enlargement distal to the terminal bronchioles [2]. Determining the presence of emphysema is important, as it has been independently associated with increased respiratory symptoms, more rapid decline in lung function, increased risk of lung cancer, higher rates of cardiovascular disease and increased mortality risk [3–6]. Surprisingly, there are some subjects with significant smoking history that have emphysema, but no airflow limitation [7]. Understanding the molecular signatures underlying emphysema may shed light on the pathogenesis of emphysema and its systemic complications.

The best current non-invasive method of detecting emphysema is high-resolution computed tomography (HRCT) [8,9]. The drawbacks to HRCT include cost, radiation exposure, and a high rate of false positive clinical significant findings (*e.g.* benign nodules); however, HRCT can provide significant information relevant to lung pathology. For instance, lung attenuation area (LAA) at −950 or −910 Hounsfield units (HU) and the mean lung attenuation value at the 15^th^ percentile (LP15A) on the lung attenuation curve are density-based measurements that correlate with emphysema [8,10,11]. Although the optimal method and normal values for describing radiologic emphysema have not been fully validated, it has been shown that control smokers without COPD have percent LAA ≤ −950 HU of <5% [9].

The first reported blood biomarker of emphysema was α1-antitrypsin (AAT); however, AAT deficiency accounts for only 1-2% of COPD [12]. Another recently reported independent biomarker of emphysema is soluble RAGE or advanced glycosylation end product receptor (AGER) [13]. Peripheral blood adiponectin and bronchoalveolar lavage fluid eotaxin levels have also correlated with radiologic emphysema [14,15]. There are other reports of peripheral blood biomarkers of airflow limitation such as interleukin-6, surfactant protein D and C-reactive protein [16,17]. Therefore, the presence of systemic biomarkers in peripheral blood, which can be easily measured and offer information regarding COPD phenotypes, may provide another method of significant value in diagnosing and managing individuals with emphysema [18]. In addition, a biomarker signature of emphysematous phenotypes may provide insight to the pathogenesis of disease. Limitations of some previous emphysema biomarker studies include small sample size and lack of replication. With this in mind, using one of the largest studies to date, we sought to determine a peripheral blood biomarker signature of emphysema, independent of other clinical variables, in current and former cigarette smokers with normal lung function and with COPD, and relate the biomarker signature to different methods of defining radiologic emphysema. Key findings were validated in an independent COPD cohort.

## Methods

### Study population

COPDGene is a multi-centered study of the genetic epidemiology of COPD that enrolled 10,192 non-Hispanic White and African-American individuals, aged 45–80 years old with at least a 10 pack-year history of smoking, who had not had an exacerbation of COPD for at least the previous 30 days. Additional information on the COPDGene study and the collection of clinical data has been described previously [19]. 1839 COPDGene subjects (1599 non-Hispanic White (NHW) and 240 non-Hispanic Black) had fresh frozen plasma collected using a p100 tube (BD) at five COPDGene sites (National Jewish Health (N =916), University of Iowa (N =670), Harbor-UCLA Medical Center (N =202), Temple University (N =36), and Baylor Medical Center (N =15)). From this cohort a subset of 602 NHW subjects (no non-Hispanic Black subjects included due to limited numbers) were selected for a comprehensive biomarker study with an attempt to obtain a range of GOLD stages and match groups as closely as possible based on age, gender and smoking history. Of the 602 subjects, 588 subjects had quantitative HRCT measurements available. The institutional review boards of participating institutions approved the study (Additional file 1: Table S1).

A separate validation cohort of 388 individuals (all former smokers with COPD) was obtained from the Treatment of Emphysema with a Selective Retinoid Agonist (TESRA) study. TESRA was a multi-centered randomized controlled trial assessing the safety and efficacy of palovarotene in ex-smokers with COPD. Only baseline samples before treatment were used for biomarker determination. Emphysema was quantitatively assessed by low dose spiral CT in the TESRA cohort. Additional information on the TESRA study has been described previously [20].

### Clinical data and definitions

COPD was defined as post bronchodilator ratio of forced expiratory volume in the first second (FEV_1_) to forced vital capacity (FVC) <0.70. Current or ex-smokers without spirometric evidence of airflow obstruction (FEV_1_/FVC ≥0.70) were classified as controls [1].

COPDGene study patients underwent whole lung volumetric multi-detector computed tomography (CT) as previously described [19,21]. Quantitative analysis of lung density was performed using the Slicer software package (http://www.slicer.org). Emphysema was primarily quantified by the percent of lung voxels (%LAA) ≤ −950 HU on the inspiratory images of CT scans for the whole lung. Emphysema was additionally quantified by percent of lung voxels (%LAA) ≤ −910 HU on inspiratory CT scans and as mean lung attenuation at the 15^th^ percentile on lung volume-adjusted attenuation curve (LP15A). In the TESRA cohort emphysema was quantified as %LAA ≤ −910 HU and LP15A on HRCT scans [20]. Densiometric analyses of the HRCTs were completed in a central lab (BioClinica, Leiden, The Netherlands) using PulmoCMS software (Medis specials, Leiden, The Netherlands). The study design and clinical outcomes have been previously reported [13,20].

### Biomarker selection and measurement

For the COPDGene cohort, 114 candidate biomarkers were selected based on a review of the literature and previously reported pilot work from the BIOSPIR group [22]. Biomarker levels were determined using a custom 15-panel assay created by Myriad-RBM (Austin, TX) multiplex technology. Blood samples were drawn from non-fasting individuals. Approximately 8.5 mL of blood was withdrawn from the ante-cubital vein into a sterile 13 × 1000 mm P100 Blood Collection Tube (BD, New Jersey, USA). The sample was immediately centrifuged at 2500 × g, 20 minutes at room temperature. Aliquots in 500 μL tubes were stored at −80°C until analyzed. In the TESRA cohort, 111 similarly chosen protein biomarkers were measured in ethylenediamine-tetraacetic acid (EDTA) plasma in duplicate at Rules Based Medicine (Austin, TX) and Quest Diagnostics (Valencia, CA). A full list of biomarkers analyzed in the TESRA study has been published [13]

### Statistical analysis

Differences in demographic characteristics of study subjects were analyzed using a *t*-test for continuous variables and a Chi-squared test for categorical variables. Emphysema severity was classified as none, mild, moderate and severe. For %LAA ≤ −950 HU the cutoffs were <5%, 5- < 10%, 10- < 20% and ≥20%, respectively, while for %LAA ≤ −910 HU the cutoffs were <35%, 35- < 45%, 45- < 55% and ≥55%, respectively. Cutoffs were based on mean values from COPDGene studies and balancing the sample size in each group [9].

Biomarkers (n = 17) with >10% and <95% of values below the lower limit of quantitation (LLOQ) for that particular biomarker were transformed into binary variables (present or absent). Biomarkers (n = 16) with >95% values below LLOQ were excluded from the analysis. For regression analysis, the remaining biomarker levels (n = 81) underwent an empirical normal quantile transformation projecting the ranks onto an inverse normal distribution so that they resemble a normal distribution and allow comparison of biomarkers at different concentrations. Non-transformed biomarker levels are also presented (Additional file 1: Table S3). Collinearity among biomarkers and covariates was assessed using Pearson correlation. Collinearity (R > 0.6) was observed between proinsulin intact (INS intact) and proinsulin total (INS total) so INS intact was removed from the analysis. Also, brain derived neurotropic factor (BDNF) was removed, as it was collinear with angiopoietin 1, CCL5 (T cell specific protein RANTES), epithelial-derived neutrophil-activating protein 78, alpha-1 antitrypsin and latency associated peptide of transforming growth factor beta 1. For modeling of multiple biomarkers, stepwise regression, with a combination of backwards and forwards selection and a p-value threshold <0.15 for entry and exit from the model, was used to arrive at the final model. A p-value of <0.05 was taken as statistically significant for association with the outcome emphysema variables.

To perform the meta-analysis, a single variable model was fit for each of the significant biomarkers that were also identified in the TESRA study. Equivalent covariates were included for the two studies and an ordered logistic and linear regression was fit respectively for the %LAA ≤ −910 HU and LP15A outcomes. P-values from both studies were combined by calculating the average Z-score of the inverse normal quantiles of the two p-values to determine a combined p-value that accounted for consistent effects of the biomarker levels on emphysema severity in the two studies [23]. A Bonferroni adjustment was applied based on all tested markers.

Receiver operating characteristic (ROC) curves were generated for covariates alone and covariates with biomarkers with the presence of emphysema compared to no emphysema as the outcome. Nominal logistic regression was performed, with emphysema considered present if %LAA ≤ −950 HU was ≥5% compared to no emphysema (%LAA ≤ −950 HU <5%). Similarly, ROC curves were generated including different severities of airflow limitation based on FEV_1_ percent predicted. Statistical analyses were performed using JMP 9.0 (SAS Institute, Cary, NC) and R (version 3.0.2) statistical software packages [24].

## Results

### Study population

Demographics, physiology, quantitative HRCT measurements and patient-reported outcomes for COPDGene and TESRA cohorts are listed in Table 1. In the COPDGene biomarker study, there were 588 individuals with complete data available. Subjects with COPD were significantly older, had lower BMI, higher pack-year history of smoking and worse SGRQ scores compared to those without COPD (p < 0.01, all comparisons). The distribution of gender and current smokers was similar between non-COPD and COPD groups. The following variables were associated with emphysema (LAA ≤ −950 HU): lower FEV_1_ (p < 0.001), lower body mass index (p < 0.001), male gender (p = 0.002), older age at enrollment (p = 0.038) and current non-smoking status (p < 0.001); these variables were used as covariates for multiple regression (Additional file 1: Table S2).

**Table 1:** Demographics of individuals in COPDGene and TESRA studies*

	**COPDGene (n = 588)**			**TESRA**
	**No COPD n = 247**	**COPD n = 341**	**p-value**	**COPD (n = 388)**
**Demographics**				
Age (years)	61 ± 3	65 ± 0.5	p < 0.01	66.6 ± 0.4
Gender (male/female)	124/123	178/163	p = 0.63	267/121
Current smokers (%)	27	23	p = 0.23	0
Smoking history (pack-years)	38 ± 1	54 ± 2	p < 0.001	48 ± 1
Body mass index (kg/m^2^)	28.9 ± 2.3	27.8 ± 0.3	p = 0.009	26 ± 0.2
**Physiology**				
FEV_1_ post bronchodilator (% predicted)	98 ± 3.6	47 ± 1	p < 0.001	50 ± 0.5
FVC post bronchodilator (% predicted)	96 ± 3.6	79 ± 1	p < 0.001	93 ± 0.9
**HRCT measurements**				
Average % LAA ≤ −950 HU	2.3 ± 1.6	15 ± 0.7	p < 0.001	N/A
% Emphysema <5%	85	31		N/A
% Emphysema 5- <10%	13	15		N/A
% Emphysema 10- <20%	2	25		N/A
% Emphysema ≥ 20%	0	29		N/A
Average % LAA ≤ −910 HU	22.6 ± 3.7	39 ± 0.7	p < 0.001	40.7 ± 0.8
% Emphysema <35%	79	35		
% Emphysema 35- <45%	15	19		
% Emphysema 45- <55%	5	19		
% Emphysema ≥55%	1	27		
Average LP15A	−916 ± 4.3	−944 ± 1.3	p < 0.001	−945 ± 1.3
**Patient-reported outcomes**				
MRC dyspnea score	0.5 ± 0.1	2.2 ± 0.1	p < 0.001	2.0 ± 0.03
SGRQ	12 ± 3.9	39 ± 1.1	p < 0.001	46 ± 0.8

*Presented are the means ± standard errors for COPDGene cohort and TESRA cohort. p values represent difference between no COPD and COPD groups for COPDGene. FEV_1_ = Forced expiratory volume at one second; FVC = forced vital capacity; LAA = low area attenuation; N/A = data not available; LP15A = mean lung attenuation value at the 15^th^ percentile on lung attenuation curve. MRC = Medical Research Council; SGRQ = St. George’s Respiratory Questionnaire.

### Biomarkers associated with emphysema

**Figure 1:**
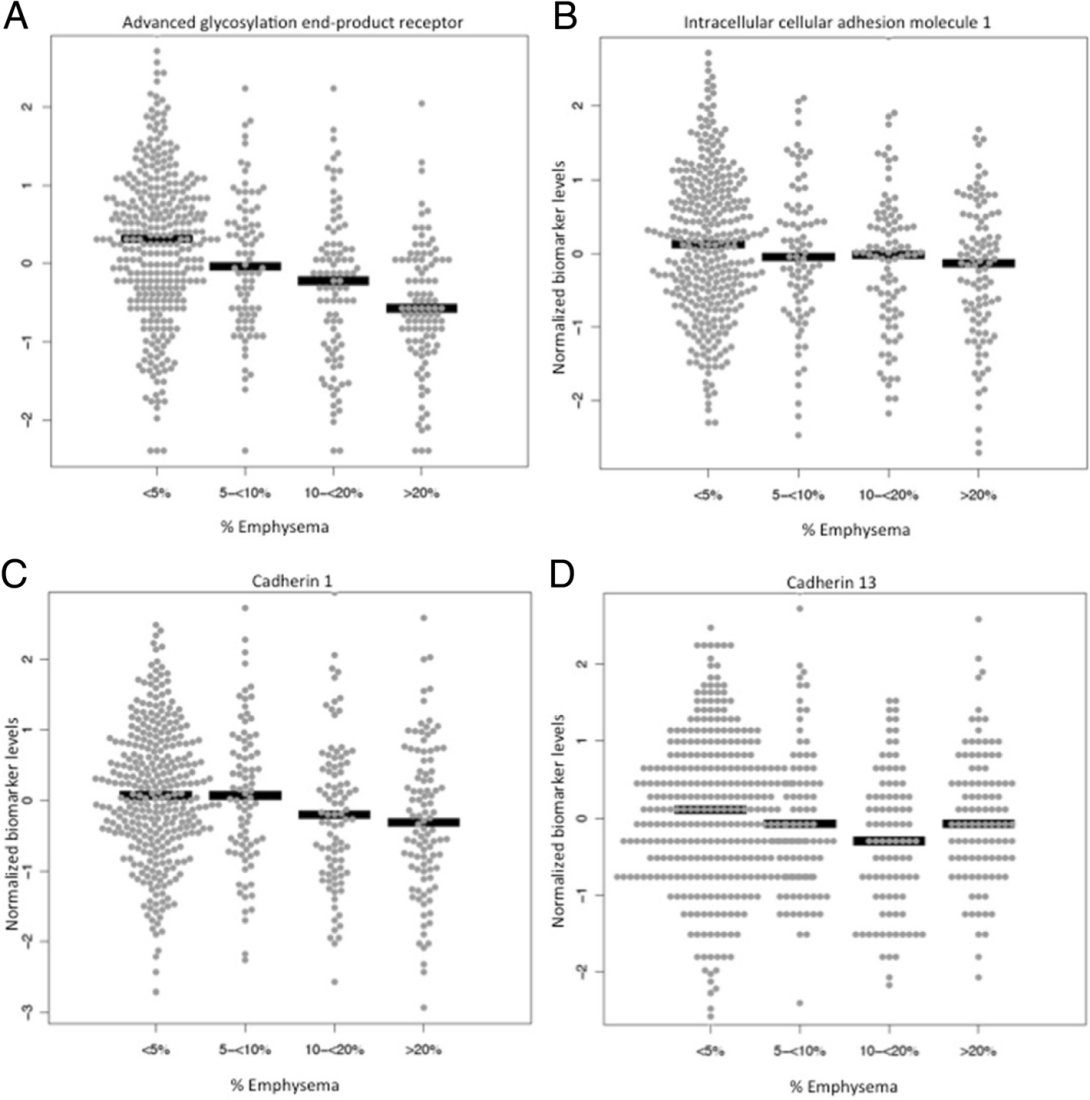
**Biomarkers associated with CT-assessed emphysema in the COPDGene cohort. (A)** Advanced glycosylation end-product receptor (RAGE); **(B)** Intracellular adhesion molecule 1 (ICAM1); **(C)** Cadherin 1 (CDH1); **(D)** Cadherin 13 (CDH13). *Presented are normal quantile transformed biomarker levels on the ordinate and percent emphysema (% low attenuation ≤ −950 HU) on CT scan on abscissa (p < 0.001 for all comparisons).

A full list of biomarkers analyzed in the COPDGene cohort is available (Additional file 1: Table S3). After adjusting for covariates, multiple regression analyses demonstrated a total of 24 biomarkers associated with radiologic emphysema including 15 biomarkers independently associated with %LAA ≤ −950 HU (R^2^ = 0.4), 9 biomarkers associated with %LAA ≤ −910 HU (R^2^ = 0.36) and 16 associated with LP15A (R^2^ = 0.64, Table 2). There were 6 biomarkers that were associated with all 3 radiologic emphysema outcome variables. Advanced glycosylation end-product receptor (RAGE) was negatively associated with more severe emphysema (Figure 1A). In addition, intercellular adhesion molecule 1 (ICAM1, Figure 1B), macrophage inhibitory protein 3a (CCL20) and cadherin 1 (CDH1, Figure 1C) were negatively associated with emphysema severity. Cadherin 13 (CDH13, Figure 1D) and thyroxin-binding globulin (SERPINA7) were positively correlated with emphysema severity (p < 0.001 for all comparisons). There were 3 biomarkers surfactant associated protein D (SFPD), FAS ligand receptor (FAS), and malondialdehyde-modified low-density lipoprotein (MDA LDL) associated with both %LAA ≤ −910 HU and LP 15 emphysema outcomes (Table 2).

**Table 2:** Biomarkers and covariates associated with radiologic emphysema in the COPDGene cohort (using multiple regression)*

	**%LAA ≤ −950 HU**		**%LAA ≤ −910 HU**		**LP15A** ^**#**^	
**Covariate**	**Beta coefficient**	**p-value**	**Beta coefficient**	**p-value**	**Beta coefficient**	**p-value**
FEV_1_ (% predicted)	−0.07	2.9 × 10^−40^	−0.05	6.4 × 10^−29^	0.42	2.1 × 10^−47^
Body mass index	−0.15	3.2 × 10^−10^	−0.26	8.2 × 10^−22^	1.37	3.4 × 10^−21^
Current active smoking	−1.16	9.1 × 10^−5^	−0.76	1.3 × 10^−7^	4.56	7.5 × 10^−7^
Male gender	0.35	0.002	0.71	7.3 × 10^−9^	−9.57	0.0001
Age at enrollment	0.04	0.039	0.04	0.006	−0.20	0.039
Biomarker						
RAGE	−0.69	2.6 × 10^−8^	−1.10	0.005	10	0.0002
CCL20 (presence)	−0.45	0.0006	−0.35	0.004	2.12	0.009
ICAM1	−0.42	0.001	−2.40	0.007	28.39	3.4 × 10^−6^
SERPINA7^**¶**^	0.28	0.013	2.11	0.042	−13.69	0.038
CDH13^**¶**^	0.29	0.025	2.62	0.005	−16.91	0.008
CDH1^**¶**^	−0.25	0.039	−2.04	0.006	13.09	0.006
TGFB1 LAP	−0.54	0.0002				
CCL13	0.35	0.013				
TNFRSF11B	0.34	0.016				
CCL8	−0.27	0.023				
IgA	−0.25	0.03			6.09	0.025
SORT1	−0.26	0.038				
IL2RA	0.27	0.044				
CCL2	0.25	0.045				
IL12B (presence)	0.22	0.049				
MDA LDL (absence)^**¶**^			0.33	0.016	−2.07	0.025
FAS			1.16	0.016	−8.53	0.014
SFTPD			−1.16	0.025	8.34	0.016
AXL					17.05	0.002
CXCL10					−11.80	0.002
ADIPOQ^**¶**^					−7.26	0.015
MB^**¶**^					−7.97	0.016
SOD1					11.08	0.009
NRCAM^**¶**^					−9.26	0.017

*Presented are beta coefficients and p values for multiple regression models of biomarkers and covariates associated with emphysema outcomes. %LAA = Percent low attenuation areas; LP15A = mean lung attenuation at 15^th^ percentile on lung attenuation curve; HU = Hounsfield units; FEV_1_ = Forced expiratory volume in 1^st^ second; RAGE = Receptor for advanced glycosylation end products; CCL20 = Macrophage Inflammatory Protein-3 alpha; ICAM1 = Intercellular Adhesion Molecule 1; SERPINA7 = Thyroxin-binding globulin; CDH 13 = Cadherin-13; CDH1 = Cadherin-1; TGFB1 LAP = Latency-Associated Peptide of Transforming Growth Factor beta 1; CCL13 = Monocyte Chemotactic Protein 4; TNFRSF11B = Osteoprotegerin; CCL8 = Monocyte Chemotactic Protein 2; IgA = Immunoglobulin A; SORT1 = Sortilin; IL2RA = Interleukin-2 receptor alpha; CCL2 = Monocyte Chemotactic Protein 1; IL-12B = Interleukin-12 Subunit p40; MDA LDL = Malondialdehyde-Modified Low-Density Lipoprotein; FAS = FASLG Receptor; SFTPD = Surfactant protein D; AXL = AXL Receptor Tyrosine Kinase; CXCL10 = Interferon gamma Induced Protein 10; ADIPOQ = Adiponectin; MB = Myoglobin; SOD1 = Superoxide dismutase 1; NRCAM = Neuronal Cell Adhesion Molecule.

^#^Higher LP15A values indicate less severe emphysema, so positive coefficients are associated with less severe emphysema and negative coefficients are associated with more severe emphysema unlike higher %LAA which is associated with more severe emphysema.

^**¶**^Biomarkers not available for replication in TESRA.

### Validation of emphysema biomarkers

Using similar statistical methods (modeling, covariates, etc.), we attempted to validate the statistically significant biomarkers using an independent cohort from the TESRA study. Although %LAA ≤ −910 HU and LP15A HRCT data were available in the TESRA cohort, %LAA ≤ −950 HU measurements were not. Therefore, of the total 16 biomarkers statistically associated with the emphysema outcomes ≤ −910 and LP15A in the COPDGene cohort, 9 biomarkers were available for validation in TESRA cohort. After meta-analysis and adjustment for multiple testing, biomarkers RAGE (p = 1.2 × 10^−9^) and ICAM1 (p = 1.5 × 10^−7^) were associated with % LAA < −910 HU (Table 3). Similarly, with regard to the LP15A emphysema outcome variable, meta-analysis with the TESRA cohort validated the association of RAGE (p = 2.5 × 10^−10^), ICAM1 (p = 6.0 × 10^−11^), and AXL (p = 3.8 × 10^−3^) with radiologic emphysema independent of covariates (Table 3). CCL20 was significantly negatively associated with emphysema in both the TESRA and COPDGene cohorts; however, meta-analysis was not possible due to CCL20 being binary in COPDGene and continuous in TESRA. Biomarkers significant in the COPDGene study such as CDH1, CDH13, SERPINA7, MDA LDL, MB, NRCAM, and ADIPOQ were not measured in the TESRA study and therefore could not be included in the meta-analysis.

**Table 3:** Meta-analysis of biomarkers associated with emphysema in COPDGene and TESRA cohorts*

	**COPDGene**		**TESRA**		**Adjusted meta-analysis p-value**
**Variable**	**Beta coefficient**	**p-value°**	**Beta coefficient**	**p-value°**	
**Percent LAA ≤ −910 HU**					
RAGE	−1.4	2.6 × 10^−5^	−0.52	9.2 × 10^−7^	1.2 × 10 ^−9^
ICAM1	−3.2	9.2 × 10^−6^	−0.37	3.4 × 10^−4^	1.5 × 10^−7^
CCL20^#^	−0.87	1.3 × 10^−4^	−0.29	2.2 × 10^−3^	N/A
**Mean lung attenuation at 15** ^**th**^ **percentile**					
RAGE	10.78	1.3 × 10^−5^	7.08	3.0 × 10^−8^	2.5 × 10 ^−10^
ICAM1	32.3	1.1 × 10^−9^	5.14	4.5 × 10^−5^	6.0 × 10^−11^
AXL	18.8	1.8 × 10^−4^	2.53	0.038	3.8 × 10^−3^
CCL20^#^	6.44	8.2 × 10^−5^	4.45	1.3 × 10^−4^	N/A

*Presented is the regression analysis for each biomarker with an adjusted meta-analysis p value. LAA = low attenuation area; RAGE = Receptor for advanced glycosylation end products; ICAM1 = Intercellular Adhesion Molecule 1; CCL20 = Macrophage Inflammatory Protein-3 alpha; AXL = AXL Receptor Tyrosine Kinase;

°p values for COPDGene and TESRA are two-sided p values.

^#^CCL20 was a binary variable in COPDGene, therefore it is the presence CCL20 that is negatively associated with emphysema in COPDGene cohort, while CCL20 was a continuous variable in TESRA also associated negatively associated with more severe emphysema. Meta-analysis was not possible given difference in variables (N/A).

ROC curves for covariates age, gender, BMI, current smoking status and FEV_1_ had an area under the curve (AUC) of 0.88 for the prediction of emphysema. ROC curves demonstrated a slight improvement in the AUC after adding 15 biomarkers to the model, raising the AUC to 0.92 (Additional file 1: Table S4 and Figure S1). However, when only considering those without severe airflow limitation (FEV_1_ ≥ 50%, n = 399), the AUC was 0.78 using covariates alone and the AUC increased to 0.85 when the biomarker panel was added to the model.

## Discussion

COPD is a phenotypically heterogeneous disease, with the presence of emphysema having implications for risk stratification and management [3–5,18]. In this study, we successfully identified and replicated a panel of peripheral blood biomarkers that was associated with emphysema independent of age, smoking status, body mass index, airflow limitation, and gender. These biomarkers (AGER, ICAM1 and CCL20) were associated with emphysema regardless of quantification technique (%LAA ≤ −950 and ≤ −910 HU and LP15A) and were replicated in an independent COPD cohort (TESRA), thus strengthening their potential utility for defining clinically relevant emphysema.

Our study reports lower RAGE levels in peripheral blood as a biomarker of increased emphysema percentage in the lungs independent of gender, age, airflow limitation, body mass index and current smoking status. RAGE (advanced glycosylation end-product receptor or AGER) is an immunoglobulin family member that is highly expressed in human lung [25]. The RAGE pathway and soluble RAGE (sRAGE), a splice variant or proteolytic cleavage product of RAGE, have been associated with several inflammatory conditions such as diabetes mellitus, vascular disease and arthritis [26,27]. The sRAGE molecule binds damaged ligands preventing these from binding to cell surface receptors and activating cell signaling pathways [28]. RAGE is active in damage-related conditions such as hyperglycemia, hypoxia, inflammation and oxidative stress [29]. While fasting blood glucose measurements were not available, 66 individuals reported a history of diabetes mellitus in the COPDGene biomarker study and there was no association between RAGE levels and self-reported history of diabetes mellitus (p = 0.88). Lower levels of sRAGE have been described in individuals with airflow limitation [30,31]. Other studies have found lower sRAGE levels associated with CT-assessed emphysema severity and cor pulmonale [32] and with CT-assessed emphysema and lower diffusing capacity of carbon monoxide using the TESRA data described in this study in combination with the ECLIPSE investigators [13]. Some studies suggest that sRAGE is increased in the lungs of patients with COPD and high levels of sRAGE may be associated with progression of emphysema [33]. Interestingly, animal studies suggest RAGE/sRAGE plays a role in alveolar development and overexpression in mouse lung leads to the development of emphysema [34]. This suggests that sRAGE, by acting as a decoy molecule, may have a different role in the developing lung and the adult lung or low sRAGE levels in COPD may result in increased inflammatory signaling in the lung.

In the present study, we found decreased ICAM1 levels correlate with increased severity of emphysema on CT scan, independent of smoking status, FEV_1_ and other covariates. ICAM1 is expressed on vascular endothelial and immune cells and mediates cell transmigration and adhesion [35]. ICAM1 plays a role in the recruitment of inflammatory cells to the lung. There is currently quite limited information about the association of ICAM1 to COPD and emphysema. Higher serum levels of soluble ICAM1 have been demonstrated in COPD, where it correlated with the severity of airflow limitation, arterial hypoxemia and hypercarbia [36,37]. Other studies relate ICAM1 levels to active smoking [38] and preliminary analysis from The MESA Lung Study demonstrated that ICAM1 predicted 0.15%/year increase in CT-assessed emphysema, suggesting a role for this molecule as a biomarker of emphysema and that it may play a role in emphysema pathogenesis [39].

CCL20 or macrophage inhibitory protein 3a, a chemokine receptor ligand, is involved in the recruitment of inflammatory cells through chemokine receptor 6 (CCR6), its only known receptor [40]. In both the COPDGene study and the TESRA study, CCL20 levels were inversely and significantly associated with emphysema although methodological considerations prevented a meta-analysis. Lower CCL20 levels have been described in bronchoalveolar lavage fluid of smokers [41]. The CCR6/CCL20 complex is one of the most potent regulators of dendritic cell migration to the lung and CCR6 knockout mice may be partially protected against cigarette smoke-induced emphysema due to reduced recruitment of inflammatory cells to the lung [42]. These data suggest that increased activity of the CCL20/CCR6 pathway may increase the susceptibility to emphysema.

CDH1 was negatively correlated with radiologic emphysema across all emphysema outcome measurements. CDH1 or E cadherin is an epithelial cell adhesion molecule that regulates cell differentiation and morphogenesis, and is associated with lung fibrosis and cancer [43]. CDH1 may be a marker of epithelial cell injury and epithelial to mesenchymal transition that is believed to play a role in small airway remodeling in COPD [44]. Genetic polymorphisms in CDH1 have been associated with development of COPD and decline in lung function [45]. CDH13 or H cadherin is another adhesion molecule that may influence surfactant protein D levels and serum adiponectin levels, both implicated in the pathogenesis of COPD; however, CDH13 itself has not been associated with quantitative emphysema to date [46,47]. We found higher levels of CDH13 to be associated with CT-assessed emphysema in the COPDGene cohort, but these were not available for validation in the TESRA cohort. Higher SERPINA7 levels were also associated with more radiologic emphysema. SERPINA7 does not have protease inhibitor capabilities and is also known as thyroid binding globulin. This study represents a new association for SERPINA7 with COPD.

With regard to the ability of biomarkers to predict the presence of any emphysema compared to no emphysema, ROC curves demonstrated a small contribution of plasma biomarkers separate to the covariates alone. This is likely because when individuals with more severe levels of emphysema are included, covariates alone, especially FEV_1_, are highly predictive of emphysema in their own right. However, the biomarkers are more useful for predicting the presence of emphysema in those that do not already have severe airflow limitation, because the covariates alone were not as good at predicting emphysema in this group and biomarkers combined with covariates increased the area under the curve. This may be useful clinically since determining the presence of underlying emphysema at this early stage in those that do not yet have severe airflow limitation may have outcome benefits for the individuals [3–6].

This COPDGene biomarker study is one of the largest emphysema biomarker studies to date on carefully phenotyped individuals with COPD. The TESRA cohort provides validation of a number of the findings. The study confirms a previously identified association between radiologic emphysema and sRAGE, builds on data suggesting a role for ICAM1 as a biomarker, in addition to discovering previously not identified biomarkers associated with emphysema such as CCL20, cadherin 1, cadherin 13 and SERPINA7. The study also highlights the potential usefulness of a panel of biomarkers to predict the presence of emphysema compared to using clinical data alone, especially in those who do not yet have severe abnormalities in lung function. However there are limitations; the TESRA cohort was different from the COPDGene cohort in that its population was comprised of ex-smokers with at least mild COPD and did not include control subjects and only 2 of the 3 quantitative emphysema measurements were made (−910 HU and LP15A). Since emphysema can occur in smokers without COPD and emphysema measurements are highly co-linear, these limitations may be of minor importance. Other limitations include the fact that the majority of subjects in both cohorts were non-Hispanic white, thus, the generalizability of these findings to other populations remains unknown and emphysema measurements from both COPDGene and TESRA were cross sectional; therefore, the significance of these biomarkers for emphysema progression remains unknown. A final limitation of this study, an in many biomarker studies, is the magnitude of association between the change in biomarker levels and the change in emphysema severity. While the biomarker associations are highly statistically significant, and validation suggests the associations are real, further studies are needed to evaluate the role of these biomarkers in disease pathogenesis and as markers of disease presence and progression [48,49].

## Conclusion

Our findings, particularly when combined with other studies of individual biomarkers, suggest that a panel of blood biomarkers including sRAGE, ICAM1 and CCL20 may serve as a useful surrogate measure of emphysema and may shed light on disease pathogenesis, providing targets for new treatments. Other biomarkers such as CHD1, CDH13 and SERPINA7 may also have a role in evaluating emphysema (especially milder emphysema), although require confirmation in other cohorts. Overall, these peripheral blood biomarkers could ultimately be used to diagnose emphysema at subclinical stages thereby reducing the need for CT, and perhaps may provide insights into disease prediction and progression.

## Additional file

Additional file 1: Table S1. Institutional Review Boards of Participating Institutions. **Table S2.** Demographics of COPDGene cohort. **Table S3.** Biomarkers in COPDGene biomarker study*. **Table S4.** Area under curve (AUC) for receiver operating characteristic (ROC) curves derived for emphysema (%LAA< -950 HU ≥5%) *vs*. no emphysema (%LAA< -950 HU <5%) as outcome*. **Figure S1.** *Receiver operating characteristic (ROC) curve with emphysema (%LAA< -950 HU ≥5%) *vs*. no emphysema (%LAA< -950 HU <5%) as outcome for **(A)** covariates age, gender, body mass index, smoking status and FEV_1_ (all ranges); **(B)** same covariates with 15 biomarkers; **(C)** covariates with FEV_1_ (≥ 50% predicted) and **(D)** covariates with FEV_1_ (≥ 50% predicted) and 15 biomarkers.

## Abbreviations

ADIPOQ: Adiponectin
AGER/RAGE: Advanced glycosylation end products receptor
AXL: AXL Receptor Tyrosine Kinase
CCL13: Monocyte chemotactic protein 4
CCL2: Monocyte chemotactic protein 1
CCL20: Macrophage inflammatory protein-3 alpha
CCL8: Monocyte chemotactic protein 2
CDH 13: Cadherin-13
CDH1: Cadherin-1
COPD: Chronic obstructive pulmonary disease
CXCL10: Interferon gamma induced protein 10
FAS: FASLG receptor
FEV1: Forced expiratory volume in first second
FVC: Forced vital capacity
HRCT: High resolution computed tomography
HU: Hounsfield units
ICAM1: Intercellular adhesion molecule 1
IgA: Immunoglobulin A
IL-12B: Interleukin-12 Subunit p40
IL2RA: Interleukin-2 receptor alpha
LAA: Low attenuation area
LLOQ: Lower limit of quantitation
LP15A: Mean lung attenuation at the 15^th^ percentile on lung attenuation curve
MB: Myoglobin
MDA LDL: Malondialdehyde-modified low-density lipoprotein
NRCAM: Neuronal cell adhesion molecule
SERPINA7: Thyroxin-binding globulin
SFTPD: Surfactant protein D
SOD1: Superoxide dismutase 1
SORT1: Sortilin
TGFB1 LAP: Latency-associated peptide of transforming growth factor beta 1
TNFRSF11B: Osteoprotegerin
