# Supplementary material for: The association of plasma biomarkers with computed tomography-assessed emphysema phenotypes
Source: Respir Res. 2014 Oct 12;15(1):127. doi: 10.1186/s12931-014-0127-9 (PMC4198701; doi:10.1186/s12931-014-0127-9)
Supplement: Additional file 1: Table S1. — Institutional Review Boards of Participating Institutions. Table S2. Demographics of COPDGene cohort. Table S3. Biomarkers in COPDGene biomarker study*. Table S4. Area under curve (AUC) for receiver operating characteristic (ROC) curves derived for emphysema (%LAA< -950 HU ≥5%) vs. no emphysema (%LAA< -950 HU <5%) as outcome*. Figure S1. *Receiver operating characteristic (ROC) curve with emphysema (%LAA< -950 HU ≥5%) vs. no emphysema (%LAA< -950 HU <5%) as outcome for (A) covariates age, gender, body mass index, smoking status and FEV1 (all ranges); (B) same covariates with 15 biomarkers; (C) covariates with FEV1 (≥ 50% predicted) and (D) covariates with FEV1 (≥ 50% predicted) and 15 biomarkers. [file 12931_2014_127_MOESM1_ESM.docx]

The association of plasma biomarkers with computed tomography-assessed emphysema phenotypes.

Brendan J. Carolan, MD^1,2^; Grant Hughes BSc^3^; Katerina Kechris, PhD^3^; Jarrett Morrow, PhD^4^; Wanda K. O’Neal, PhD^5^; Stephen Rennard, MD^6^; Sreekumar G. Pillai PhD^7^; Paula Belloni PhD^8^; Debra A. Cockayne, PhD^9^; Alejandro P. Comellas, MD^10^; Meilan Han, MD^11^; Craig P. Hersh MD^4^; Russell P. Bowler MD, PhD^1,2^

**Additional file**

**Table S1. Institutional Review Boards of Participating Institutions**

| **Clinical Center** | **Institution Title** | **Protocol Number** |
| --- | --- | --- |
| National Jewish Health | National Jewish IRB | HS-1883a |
| Brigham and Women’s Hospital | Partners Human Research Committee | 2007-P-000554/2; BWH |
| Baylor College of Medicine | Institutional Review Board for Baylor  College of Medicine and Affiliated Hospitals | H-22209 |
| Michael E. DeBakey VAMC | Institutional Review Board for Baylor College of Medicine and Affiliated Hospitals | H-22202 |
| Columbia University Medical Center | Columbia University Medical Center IRB | IRB-AAAC9324 |
| Duke University Medical Center | The Duke University Health System Institutional Review Board for Clinical Investigations (DUHS IRB) | Pro00004464 |
| Johns Hopkins University | Johns Hopkins Medicine Institutional Review Boards (JHM IRB) | NA_00011524 |
| Los Angeles Biomedical Research Institute | The John F. Wolf, MD Human Subjects Committee of Harbor-UCLA Medical Center | 12756-01 |
| Morehouse School of Medicine | Morehouse School of Medicine Institutional Review Board | 07-1029 |
| Temple University | Temple University Office for Human Subjects Protections Institutional Review Board | 11369 |
| University of Alabama at Birmingham | The University of Alabama at Birmingham Institutional Review Board for Human Use | FO70712014 |
| University of California, San Diego | University of California, San Diego Human Research Protections Program | 070876 |
| University of Iowa | The University of Iowa Human Subjects Office | 200710717 |
| Ann Arbor VA | VA Ann Arbor Healthcare System IRB | PCC 2008-110732 |
| University of Minnesota | University of Minnesota Research Subjects’ Protection Programs (RSPP) | 0801M24949 |
| University of Pittsburgh | University of Pittsburgh Institutional Review Board | PRO07120059 |
| University of Texas Health Sciences Center at San Antonio | UT Health Science Center San Antonio Institutional Review Board | HSC20070644H |
| Health Partners Research Foundation | Health Partners Research Foundation Institutional Review Board | 07-127 |
| University of Michigan | Medical School Institutional Review Board (IRBMED) | HUM00014973 |
| Minneapolis VA Medical Center | Minneapolis VAMC IRB | 4128-A |
| Reliant Clinic | Institutional Review Board/Research Review Committee Saint Vincent Hospital – Fallon Clinic – Fallon Community Health Plan | 1143 |

**Table S2.** Demographics of COPDGene cohort.

|  | **COPDGene (n=588)** | | | | | |
| --- | --- | --- | --- | --- | --- | --- |
|  | **<5% LAA <-950 HU**  **(n=315)** | ≥**5% LAA <-950 HU**  **(n=273)** | | | |  |
|  |  | 5-10%  (n=82) | 10-20%  (n=91) | >20%  (n=100) | Total  ≥5% | p Value  (<%5 *vs*. ≥5%) |
| **Demographics** |  |  |  |  |  |  |
| Age (years) | 61 ± 0.5 | 64 ± 1 | 67 ± 0.7 | 66 ± 0.8 | 66 ± 0.5 | p<0.01 |
| Gender (male/female) | 142/173 | 49/33 | 52/39 | 59/41 | 160/113 | p<0.001 |
| Current smokers (%) | 33 | 29 | 12 | 8 | 16 | p<0.01 |
| Smoking History (pack-years) | 42 ± 1 | 50 ± 3 | 56 ± 3 | 53 ± 3 | 53 ± 2 | p<0.01 |
| Body mass index (kg/m^2^) | 29.5 ± 0.3 | 28.6 ± 0.6 | 28.2 ± 0.5 | 24 ± 0.4 | 26.9 ± 0.3 | P<0.001 |
| **Physiology** |  |  |  |  |  |  |
| FEV_1_ post bronchodilator (% predicted) | 85 ± 1.2 | 70 ± 3.2 | 47± 2.1 | 35 ± 1.3 | 49 ± 1.5 | P<0.001 |
| FVC post bronchodilator (% predicted) | 90 ± 0.9 | 87 ± 2.1 | 81 ± 2.0 | 79 ± 2.2 | 82 ± 1.2 | P<0.001 |
| COPD by GOLD (%) | 33 | 61 | 94 | 100 | 86 | P<0.001 |
| **HRCT measurements** |  |  |  |  |  |  |
| %Emphysema Total lung (-950 HU) | 1.6 ± 0.07 | 7.2 ± 0.2 | 14.8 ± 0.3 | 31.7 ± 0.8 | 18 ± 0.7 | P<0.001 |
| % Emphysema Total lung (-910 HU) | 19.4 ± 0.6 | 39 ± 0.9 | 45.6 ± 0.8 | 60.4 ± 0.7 | 39 ± 0.7 | P<0.001 |
| Emphysema Total lung (LP15A) | -913 ± 0.9 | -937 ± 0.5 | -951 ± 0.5 | -972 ± 0.9 | -954 ± 0.9 | P<0.001 |
| **Patient-reported outcomes** |  |  |  |  |  |  |
| MRC dyspnea score | 1.0 ± 0.1 | 1.5 ± 0.2 | 2.0 ± 0.1 | 2.9 ± 0.1 | 2.2 ± 0.1 | P<0.001 |
| SGRQ | 19 ± 1.2 | 29 ± 2.7 | 38 ± 2.1 | 47 ± 1.6 | 38 ± 1.3 | P<0.001 |

*Presented are the means ± standard errors for COPDGene cohort and TESRA cohort. LAA = low area attenuation; FEV_1_ =Forced expiratory volume at one second; FVC = forced vital capacity; % Emphysema = % low area attenuation < -950 HU and <-910 HU on inspiration; LP15A = mean lung attenuation value at the 15^th^ percentile on lung attenuation curve. MRC =Medical Research Council; SGRQ =St. George’s Respiratory Questionnaire; p values represent difference between <5% emphysema (%LAA<-950 HU) group and ≥5% emphysema group.

**Table S3:** Biomarkers in COPDGene biomarker study*.

| **Biomarker abbreviation** | **Biomarker name** | **Units** | **Variable type** | **25th Percentile** | **Median** | **75^th^ Percentile** | **LLOQ** | **% below LLOQ** |
| --- | --- | --- | --- | --- | --- | --- | --- | --- |
| A2M | Alpha-2-Macroglobulin (A2Macro) | mg/mL | Continuous | 0.94 | 1 | 1.2 | 0.023 | 0% |
| ADIPO | Adiponectin | ug/mL | Continuous | 3.5 | 5.3 | 8.05 | 0.051 | 0% |
| APCS | Serum Amyloid P-Component (SAP) | ug/mL | Continuous | 14 | 17 | 21 | 0.093 | 0% |
| APOA4 | Apolipoprotein A-IV (Apo A-IV) | ug/mL | Continuous | 66 | 167 | 459.5 | 0.96 | 0% |
| AXL | AXL Receptor Tyrosine Kinase (AXL) | ng/mL | Continuous | 9.7 | 12 | 15 | 0.050 | 0% |
| B2M | Beta-2-Microglobulin (B2M) | ug/mL | Continuous | 1.4 | 1.8 | 2.3 | 0.0069 | 0% |
| C3 | Complement C3 (C3) | mg/mL | Continuous | 1.1 | 1.2 | 1.4 | 0.020 | 0% |
| CCL16 | Chemokine CC-4 (HCC-4) | ng/mL | Continuous | 4 | 5.3 | 7 | 0.047 | 0% |
| CCL18 | Pulmonary and Activation-Regulated Chemokine (PARC) | ng/mL | Continuous | 63 | 89 | 119.5 | 6.2 | 0% |
| CCL22 | Macrophage-Derived Chemokine (MDC) | pg/mL | Continuous | 308.5 | 400 | 487.75 | 19 | 0% |
| CCL23 | Myeloid Progenitor Inhibitory Factor 1 (MPIF-1) | ng/mL | Continuous | 1.1 | 1.4 | 1.7 | 0.18 | 0% |
| CCL24 | Eotaxin-2 | pg/mL | Continuous | 359 | 553 | 844 | 50 | 0% |
| CCL4 | Macrophage Inflammatory Protein-1 beta (MIP-1 beta) | pg/mL | Continuous | 152.25 | 197 | 255 | 31 | 0% |
| CCL5 | T-Cell-Specific Protein RANTES (RANTES) | ng/mL | Continuous | 5.25 | 9.8 | 17 | 0.024 | 0% |
| CDH1 | Cadherin-1 (E-Cad) | ng/mL | Continuous | 2570 | 3110 | 4000 | 6.0 | 0% |
| CDH13 | Cadherin-13 (T-cad) | ng/mL | Continuous | 15 | 18 | 22 | 2.2 | 0% |
| CEACAM1 | Carcinoembryonic antigen-related cell adhesion molecule 1 (CEACAM1) | ng/mL | Continuous | 12 | 14 | 16 | 3.1 | 0% |
| CHGA | Chromogranin-A (CgA) | ng/mL | Continuous | 334.25 | 485.5 | 774.75 | 13 | 0% |
| CRP | C-Reactive Protein (CRP) | ug/mL | Continuous | 1.3 | 2.7 | 6 | 0.048 | 0% |
| CSTB | Cystatin-B | ng/mL | Continuous | 7.3 | 9.4 | 12 | 0.34 | 0% |
| CXCL10 | Interferon gamma Induced Protein 10 (IP-10) | pg/mL | Continuous | 206 | 262 | 354 | 100 | 0% |
| CXCL9 | Monokine Induced by Gamma Interferon (MIG) | pg/mL | Continuous | 697.5 | 1020 | 1550 | 143 | 0% |
| DCN | Decorin | ng/mL | Continuous | 1.7 | 1.9 | 2.2 | 0.13 | 0% |
| F7 | Factor VII | ng/mL | Continuous | 456 | 563 | 688 | 1.8 | 0% |
| FTL_FTH1 | Ferritin (FRTN) | ng/mL | Continuous | 66 | 120 | 217.5 | 4.3 | 0% |
| GC | Vitamin D-Binding Protein (VDBP) | ug/mL | Continuous | 219.5 | 278 | 348.5 | 5.6 | 0% |
| ICAM1 | Intercellular Adhesion Molecule 1 (ICAM-1) | ng/mL | Continuous | 102 | 125 | 151 | 1.5 | 0% |
| IgM | Immunoglobulin M (IgM) | mg/mL | Continuous | 1.05 | 1.6 | 2.3 | 0.094 | 0% |
| IL16 | Interleukin-16 (IL-16) | pg/mL | Continuous | 330.25 | 393 | 464.75 | 87 | 0% |
| IL18BP | Interleukin-18-binding protein (IL-18bp) | ng/mL | Continuous | 9.1 | 11 | 14 | 0.096 | 0% |
| IL2RA | Interleukin-2 receptor alpha (IL-2 receptor alpha) | pg/mL | Continuous | 1712.5 | 2100 | 2667.5 | 420 | 0% |
| IL6R | Interleukin-6 receptor (IL-6r) | ng/mL | Continuous | 23 | 28 | 34 | 0.018 | 0% |
| KIT | Mast/stem cell growth factor receptor (SCFR) | ng/mL | Continuous | 6.8 | 8.1 | 9.3 | 0.51 | 0% |
| MB | Myoglobin | ng/mL | Continuous | 24.5 | 34 | 48 | 2.1 | 0% |
| MMP3 | Matrix Metalloproteinase-3 (MMP-3) | ng/mL | Continuous | 5.6 | 8.2 | 12 | 0.049 | 0% |
| PECAM1 | Platelet endothelial cell adhesion molecule (PECAM-1) | ng/mL | Continuous | 37 | 44 | 52 | 11 | 0% |
| SELE | E-Selectin | ng/mL | Continuous | 5.6 | 7.6 | 10 | 0.31 | 0% |
| SERPINA1 | Alpha-1-Antitrypsin (AAT) | mg/mL | Continuous | 1.6 | 1.8 | 2.1 | 0.016 | 0% |
| SERPINA7 | Thyroxine-Binding Globulin (TBG) | ug/mL | Continuous | 32 | 37 | 42 | 0.22 | 0% |
| SFTPD | Pulmonary surfactant-associated protein D (SP-D) | ng/mL | Continuous | 4.7 | 6.6 | 8.8 | 0.19 | 0% |
| SHBG | Sex Hormone-Binding Globulin (SHBG) | nmol/L | Continuous | 39 | 54 | 76.5 | 3.1 | 0% |
| SLPI | Antileukoproteinase (ALP) | ng/mL | Continuous | 33 | 37 | 42 | 0.98 | 0% |
| SOD1 | Superoxide Dismutase 1, soluble (SOD-1) | ng/mL | Continuous | 25 | 32 | 41 | 0.12 | 0% |
| SORT1 | Sortilin | ng/mL | Continuous | 4.9 | 5.8 | 6.9 | 0.22 | 0% |
| SPINK1 | Pancreatic secretory trypsin inhibitor (TATI) | ng/mL | Continuous | 10 | 13 | 18 | 0.16 | 0% |
| TGFB1_LAP | Latency-Associated Peptide of Transforming Growth Factor beta 1 (LAP TGF-b1) | ng/mL | Continuous | 2.5 | 3.8 | 6.2 | 0.13 | 0% |
| THBD | Thrombomodulin (TM) | ng/mL | Continuous | 3.8 | 4.4 | 5.2 | 0.052 | 0% |
| TIMP1 | Tissue Inhibitor of Metalloproteinases 1 (TIMP-1) | ng/mL | Continuous | 63 | 72 | 86.5 | 1.2 | 0% |
| TIMP2 | Tissue Inhibitor of Metalloproteinases 2 (TIMP-2) | ng/mL | Continuous | 59 | 66 | 73 | 1.5 | 0% |
| TNFRSF10C | TNF-Related Apoptosis-Inducing Ligand Receptor 3 (TRAIL-R3) | ng/mL | Continuous | 9.1 | 13 | 17 | 0.96 | 0% |
| TNFRSF11B | Osteoprotegerin (OPG) | pM | Continuous | 4.5 | 5.5 | 6.6 | 0.45 | 0% |
| TNFRSF1A | Tumor Necrosis Factor Receptor I (TNF RI) | pg/mL | Continuous | 1350 | 1630 | 2017.5 | 36 | 0% |
| TNFRSF1B | Tumor necrosis factor receptor 2 (TNFR2) | ng/mL | Continuous | 4.6 | 5.6 | 7.1 | 0.86 | 0% |
| VCAM1 | Vascular Cell Adhesion Molecule-1 (VCAM-1) | ng/mL | Continuous | 430.5 | 505 | 598 | 2.4 | 0% |
| FGA_FGB_FGG | Fibrinogen | mg/mL | Continuous | 3.6 | 4.2 | 4.8 | 0.049 | 0.17% |
| IL18 | Interleukin-18 (IL-18) | pg/mL | Continuous | 169 | 229 | 301.75 | 41 | 0.17% |
| LPA | Apolipoprotein(a) (Lp(a)) | ug/mL | Continuous | 6.7 | 7.8 | 9.1 | 1.6 | 0.17% |
| MMP9 | Matrix Metalloproteinase-9 (MMP-9) | ng/mL | Continuous | 201 | 299 | 451 | 37 | 0.17% |
| NPPB_PH | N-terminal prohormone of brain natriuretic peptide (NT proBNP) | pg/mL | Continuous | 235 | 460 | 838 | 16 | 0.17% |
| NRCAM | Neuronal Cell Adhesion Molecule (Nr-CAM) | ng/mL | Continuous | 0.695 | 0.9 | 1.2 | 0.20 | 0.17% |
| SERPINA3 | Alpha-1-Antichymotrypsin (AACT) | ug/mL | Continuous | 664.5 | 748 | 861 | 13 | 0.17% |
| CCL2 | Monocyte Chemotactic Protein 1 (MCP-1) | pg/mL | Continuous | 113 | 139 | 175.75 | 45 | 0.33% |
| CCL8 | Monocyte Chemotactic Protein 2 (MCP-2) | pg/mL | Continuous | 21 | 27 | 33.5 | 8.6 | 0.33% |
| IgA | Immunoglobulin A (IgA) | mg/mL | Continuous | 1.4 | 2 | 2.8 | 0.056 | 0.33% |
| ANGPT1 | Angiopoietin-1 (ANG-1) | ng/mL | Continuous | 5.3 | 7 | 9.3 | 2.1 | 0.50% |
| BDNF | Brain-Derived Neurotrophic Factor (BDNF) | ng/mL | Continuous | 1.5 | 3 | 5.5 | 0.062 | 0.50% |
| CKM_CKB | Creatine Kinase-MB (CK-MB) | ng/mL | Continuous | 0.97 | 1.4 | 2.1 | 0.35 | 0.50% |
| MDK | Midkine | ng/mL | Continuous | 1.6 | 2 | 2.5 | 0.46 | 0.66% |
| KITLG | Stem Cell Factor (SCF) | pg/mL | Continuous | 237 | 301 | 367 | 119 | 0.83% |
| SERPINE1 | Plasminogen Activator Inhibitor 1 (PAI-1) | ng/mL | Continuous | 21 | 34 | 49.5 | 2.8 | 0.83% |
| CXCL5 | Epithelial-Derived Neutrophil-Activating Protein 78 (ENA-78) | ng/mL | Continuous | 0.39 | 0.7 | 1.2 | 0.084 | 1.00% |
| VWF | von Willebrand Factor (vWF) | ug/mL | Continuous | 58 | 77 | 102 | 25 | 1.16% |
| RAGE | Receptor for advanced glycosylation end products (RAGE) | ng/mL | Continuous | 1.4 | 2.2 | 3.6 | 0.35 | 1.33% |
| HGF | Hepatocyte Growth Factor (HGF) | ng/mL | Continuous | 4.1 | 5.6 | 6.8 | 1.0 | 1.33% |
| IL8 | Interleukin-8 (IL-8) | pg/mL | Continuous | 7.3 | 9.4 | 13 | 4.0 | 1.33% |
| VEGFA | Vascular Endothelial Growth Factor (VEGF) | pg/mL | Continuous | 92 | 115 | 149 | 50 | 1.33% |
| HP | Haptoglobin | mg/mL | Continuous | 0.935 | 1.4 | 2 | 0.064 | 1.49% |
| CCL13 | Monocyte Chemotactic Protein 4 (MCP-4) | pg/mL | Continuous | 1340 | 1640 | 2130 | 972 | 2.65% |
| FAS | FASLG Receptor (FAS) | ng/mL | Continuous | 11 | 15 | 20 | 5.8 | 3.48% |
| LTF | Lactoferrin (LTF) | ng/mL | Continuous | 10 | 14 | 19 | 6.4 | 4.15% |
| IFNG | Interferon gamma (IFN-gamma) | pg/mL | Continuous | 2.3 | 3.2 | 4.3 | 1.5 | 8.62% |
| IL12B | Interleukin-12 Subunit p40 (IL-12p40) | ng/mL | Binary | NA | NA | NA | 0.22 | 38.31% |
| CA9 | Carbonic anhydrase 9 (CA-9) | ng/mL | Binary | NA | NA | NA | 0.22 | 43.28% |
| IgE | Immunoglobulin E (IgE) | U/mL | Binary | NA | NA | NA | 18 | 43.62% |
| MICA | MHC class I chain-related protein A (MICA) | pg/mL | Binary | NA | NA | NA | 73 | 45.94% |
| KLK3_F | Prostate-Specific Antigen, Free (PSA-f) | ng/mL | Binary | NA | NA | NA | 0.013 | 49.75% |
| CCL11 | Eotaxin-1 | pg/mL | Binary | NA | NA | NA | 144 | 56.38% |
| IL15 | Interleukin-15 (IL-15) | ng/mL | Binary | NA | NA | NA | 0.39 | 58.37% |
| CCL20 | Macrophage Inflammatory Protein-3 alpha (MIP-3 alpha) | pg/mL | Binary | NA | NA | NA | 38 | 73.13% |
| IL23A | Interleukin-23 (IL-23) | ng/mL | Binary | NA | NA | NA | 0.68 | 75.95% |
| IL1RN | Interleukin-1 receptor antagonist (IL-1ra) | pg/mL | Binary | NA | NA | NA | 220 | 76.62% |
| MDA_LDL | Malondialdehyde-Modified Low-Density Lipoprotein (MDA-LDL) | ng/mL | Binary | NA | NA | NA | 22 | 82.42% |
| INS_intact | Proinsulin, Intact | pM | Binary | NA | NA | NA | 7.1 | 83.75% |
| INS_total | Proinsulin, Total | pM | Binary | NA | NA | NA | 34 | 83.75% |
| CCL3 | Macrophage Inflammatory Protein-1 alpha (MIP-1 alpha) | pg/mL | Binary | NA | NA | NA | 42 | 84.74% |
| IL10 | Interleukin-10 (IL-10) | pg/mL | Binary | NA | NA | NA | 6.9 | 90.55% |
| IL17A | Interleukin-17 (IL-17) | pg/mL | Binary | NA | NA | NA | 2.9 | 94.03% |
| OLR1 | Lectin-Like Oxidized LDL Receptor 1 (LOX-1) | ng/mL | Binary | NA | NA | NA | 0.75 | 94.53% |
| IL1A | Interleukin-1 alpha (IL-1 alpha) | ng/mL | Excluded | NA | NA | NA | 0.0012 | 95.52% |
| TNF | Tumor Necrosis Factor alpha (TNF-alpha) | pg/mL | Excluded | NA | NA | NA | 23 | 97.68% |
| IL6 | Interleukin-6 (IL-6) | pg/mL | Excluded | NA | NA | NA | 11 | 98.18% |
| HSPD1 | Heat Shock Protein 60 (HSP-60) | ng/mL | Excluded | NA | NA | NA | 45 | 98.34% |
| LTA | Tumor Necrosis Factor beta (TNF-beta) | pg/mL | Excluded | NA | NA | NA | 9.7 | 98.51% |
| IL1B | Interleukin-1 beta (IL-1 beta) | pg/mL | Excluded | NA | NA | NA | 4.8 | 98.67% |
| IL2 | Interleukin-2 (IL-2) | pg/mL | Excluded | NA | NA | NA | 8.3 | 98.84% |
| IL7 | Interleukin-7 (IL-7) | pg/mL | Excluded | NA | NA | NA | 8.8 | 98.84% |
| IL13 | Interleukin-13 (IL-13) | pg/mL | Excluded | NA | NA | NA | 6.2 | 99.17% |
| IL12A_IL12B | Interleukin-12 Subunit p70 (IL-12p70) | pg/mL | Excluded | NA | NA | NA | 38 | 99.34% |
| IL4 | Interleukin-4 (IL-4) | pg/mL | Excluded | NA | NA | NA | 29 | 99.34% |
| CSF2 | Granulocyte-Macrophage Colony-Stimulating Factor (GM-CSF) | pg/mL | Excluded | NA | NA | NA | 88 | 99.67% |
| IL3 | Interleukin-3 (IL-3) | ng/mL | Excluded | NA | NA | NA | 0.016 | 99.83% |
| IL5 | Interleukin-5 (IL-5) | pg/mL | Excluded | NA | NA | NA | 13 | 99.83% |
| NGF | Nerve Growth Factor beta (NGF-beta) | ng/mL | Excluded | NA | NA | NA | 0.078 | 100% |
| S100B | S100 calcium-binding protein B (S100-B) | ng/mL | Excluded | NA | NA | NA | 0.50 | 100% |

***** Presented is the full list of biomarkers measured in COPDGene cohort subjects. LLOQ = lower limit of quantification. Biomarkers treated as continuous variables were transformed by quantile normalization. Biomarkers with more than 10% and less than 95% of values below LLOQ were transformed into binary variables (present or absent). Biomarkers with > 95% values below LLOQ were excluded from the analysis. Median values for raw measurements together with 25^th^ and 75^th^ percentiles are presented for continuous variables.

**Table S4**

**Area under curve (AUC) for receiver operating characteristic (ROC) curves derived for emphysema (%LAA< -950 HU ≥5%) *vs*. no emphysema (%LAA< -950 HU <5%) as outcome*.**

| **Outcome: Emphysema yes (≥5%) : no (<5%)** |  |
| --- | --- |
| Covariates with FEV_1_ (including all ranges of airflow limitation, n=588) | AUC |
| Age, gender, BMI, smoking status | 0.72 |
| Age, gender, BMI, smoking status, FEV_1_ (all ranges) | 0.88 |
| Age, gender, BMI, smoking status, FEV_1_ (all ranges), 15 biomarkers | 0.92 |
| Covariates with FEV_1_ (excluding severe and very severe airflow limitation, n=399) | AUC |
| Age, gender, BMI, smoking status, FEV_1_ (≥50% predicted) | 0.78 |
| Age, gender, BMI, smoking status, FEV_1_ (≥50% predicted), and 15 biomarkers | 0.85 |
| Covariates with FEV_1_ (including only severe and very severe airflow limitation, n=189) | AUC |
| Age, gender, BMI, smoking status, FEV_1_ (<50% predicted) | 0.86 |
| Age, gender, BMI, smoking status, FEV_1_ (<50% predicted) and 15 biomarkers | 0.93 |

*Presented is the area under the curve (AUC) for receiver operating characteristic curves derived for the presence of emphysema compared to no emphysema on CT scan for all individuals and separately for those without severe airflow limitation and those with severe airflow limitation. BMI= Body mass index; FEV1= Forced expiratory volume in 1^st^ second.

**Figure S1.***

**Receiver operating characteristic (ROC) curve with emphysema (%LAA< -950 HU ≥5%) *vs*. no emphysema (%LAA< -950 HU <5%) as outcome for (A) covariates age, gender, body mass index, smoking status and FEV_1_ (all ranges); (B) same covariates with 15 biomarkers; (C) covariates with FEV_1_ (≥ 50% predicted) and (D) covariates with FEV_1_ (≥ 50% predicted) and 15 biomarkers**

**A**. Covariates with FEV_1_ **B.** Covariates with FEV_1_ and 15 biomarkers

AUC 0.88 AUC 0.92

**C.** Covariates with FEV_1_ (≥50% predicted) **D.** Covariates with FEV_1_ (≥50% predicted) and 15 biomarkers

AUC 0.78 AUC 0.85

*Presented are ROC curves for covariates age, gender, body mass index, current smoking status with FEV_1_ (all ranges and excluding severe and very severe airflow limitation) with and without 15 biomarkers from the multiple regression model (RAGE, ICAM1, CCL20, SERPINA7, CDH13, CDH1, TGFB1 LAP, CCL13, TNFRSF11B, CCL8, IgA, SORT1, IL2RA, CCL2, IL12B) as labeled A-D. Nominal logistic regression was performed to derive the ROC curves with emphysema compared to no emphysema as the outcome. Emphysema was considered present if %LAA <-950 HU was ≥5% and emphysema was absent if %LAA <-950 HU <5%. AUC = Area under curve.
